# DNA barcoding of Northern Nearctic Muscidae (Diptera) reveals high correspondence between morphological and molecular species limits

**DOI:** 10.1186/1472-6785-12-24

**Published:** 2012-11-23

**Authors:** Anaïs K Renaud, Jade Savage, Sarah J Adamowicz

**Affiliations:** 1 Department of Entomology, University of Manitoba, Winnipeg, MB, R3T 2N2, Canada; 2 Department of Biological Sciences, Bishop’s University, Sherbrooke, Québec J1M 1Z7, Canada; 3 Biodiversity Institute of Ontario & Department of Integrative Biology, University of Guelph, Guelph, ON, N1G 2W1, Canada

**Keywords:** Insects, Muscid flies, Churchill, Manitoba, Barcoding biotas, Cytochrome *c* oxidase subunit 1, COI, DNA barcoding, Clustering-based method, Threshold-based method

## Abstract

**Background:**

Various methods have been proposed to assign unknown specimens to known species using their DNA barcodes, while others have focused on using genetic divergence thresholds to estimate “species” diversity for a taxon, without a well-developed taxonomy and/or an extensive reference library of DNA barcodes. The major goals of the present work were to: a) conduct the largest species-level barcoding study of the Muscidae to date and characterize the range of genetic divergence values in the northern Nearctic fauna; b) evaluate the correspondence between morphospecies and barcode groupings defined using both clustering-based and threshold-based approaches; and c) use the reference library produced to address taxonomic issues.

**Results:**

Our data set included 1114 individuals and their COI sequences (951 from Churchill, Manitoba), representing 160 morphologically-determined species from 25 genera, covering 89% of the known fauna of Churchill and 23% of the Nearctic fauna. Following an iterative process through which all specimens belonging to taxa with anomalous divergence values and/or monophyly issues were re-examined, identity was modified for 9 taxa, including the reinstatement of *Phaonia luteva* (Walker) **stat. nov.** as a species distinct from *Phaonia errans* (Meigen). In the post-reassessment data set, no distinct gap was found between maximum pairwise intraspecific distances (range 0.00-3.01%) and minimum interspecific distances (range: 0.77-11.33%). Nevertheless, using a clustering-based approach, all individuals within 98% of species grouped with their conspecifics with high (>95%) bootstrap support; in contrast, a maximum species discrimination rate of 90% was obtained at the optimal threshold of 1.2%. DNA barcoding enabled the determination of females from 5 ambiguous species pairs and confirmed that 16 morphospecies were genetically distinct from named taxa. There were morphological differences among all distinct genetic clusters; thus, no cases of cryptic species were detected.

**Conclusions:**

Our findings reveal the great utility of building a well-populated, species-level reference barcode database against which to compare unknowns. When such a library is unavailable, it is still possible to obtain a fairly accurate (within ~10%) rapid assessment of species richness based upon a barcode divergence threshold alone, but this approach is most accurate when the threshold is tuned to a particular taxon.

## Background

Species are generally considered the vital “currency of biodiversity” research [1]. Since taxonomic knowledge and identification tools are still weak or absent for many groups, especially in the hyper-diverse Arthropoda [2-4], the last decades have seen a sharp increase in the integration of genetic data such as DNA barcodes [5] into the invertebrate biodiversity research workflow [6-10]. DNA-based identification of a specimen, as contrasted with “DNA taxonomy” [11], relies on the comparison of its DNA barcode with those of determined individuals [5]. The collaboration of experts is therefore required to develop such reference libraries, which remain poorly populated for most insects, and to test the ability of DNA barcodes to discriminate among species, whose boundaries are defined based on morphology or integrative approaches (e.g. incorporating morphological, genetic, and ecological data [12-15]). Several methods have been proposed to assign unknown individuals to known species based on their DNA barcodes, including calculating bootstrap values to determine cluster support (e.g. [16]); pinpointing diagnostic genetic characters to separate and identify members of closely related species (e.g. [17]); and comparing genetic divergences between unknown and reference sequences to a threshold that typically differentiates intraspecific versus interspecific matches [18]. All three methods are expected to perform well if there is a taxonomically well-characterized and well-populated reference database [19,20]. However, given that perhaps only 5-10% of animal species are described [4,21] and that rare taxa are commonly encountered in biodiversity research [22], approaches that do not rely upon a well-developed taxonomy would be valuable for accelerating biodiversity research, such as flagging individuals that require taxonomic attention, and for performing rapid biodiversity inventories. Therefore, increased attention to the possibility of threshold-based approaches is warranted despite criticisms of thresholds (e.g. [19,23-25]). While a threshold of approximately 2% was originally suggested for congeneric species in most invertebrate taxa [18], the success of threshold-based approaches does not rely upon finding a single universal threshold as different values could be applied to different higher taxa, depending upon their rates of speciation and molecular evolution. Moreover, relaxed clustering methods that permit larger divergences within cohesive clusters may give even greater utility to distance-based approaches.

Previous reports of high failure rates using DNA barcoding thresholds have often relied upon identifications obtained from sequence databases (e.g. [23]). Obtaining test datasets in this way is particularly worrisome as there is no way to revisit identifications in light of unexpected results. Regarding such identifications as *facts* against which to *test* barcoding is therefore problematic. Other reported cases of high failure rates using traditional morphospecies definitions were largely resolved upon using “evolutionary significant units” [19] instead of morphospecies, suggesting that some morphospecies may require taxonomic revision. Many other studies that have investigated thresholds concluded that high intraspecific divergences are likely to reflect the presence of cryptic or new species while low divergences may reflect hybridization [26,27], but most do not conduct formal morphological re-analysis of such cases.

The ideal scenario for testing DNA barcoding would involve the following conditions: a) selection of a taxonomically well-studied group, b) identifications performed by the same team and cross-validated by one person for consistency, c) vouchers retained for all individuals to allow re-analysis, d) re-examination of identifications in light of integrative consideration of joint evidence regarding the most likely “true” species boundaries (morphology, genetics, biogeography, ecology), and e) inclusion of individuals from multiple geographic regions [19,28,29] to gain more accurate information regarding maximum levels of intraspecific variability. The present study, on the Diptera family Muscidae, meets all of these conditions. Moreover, this study contributes valuable new information about DNA barcode diversity in an understudied yet hyperdiverse insect order.

The Diptera family Muscidae is a large and ecologically diverse taxon containing over 5210 species worldwide [30] and at least 700 in the Nearctic region [31]. Muscid flies can be found in a broad range of terrestrial and aquatic habitats, but they are especially diverse and abundant in northern and alpine environments. In northern Canada and Alaska, they represent about one-quarter of all Diptera species and close to 10% of overall insect diversity [32]. Adults can be saprophagous, predaceous, haematophagous, or anthophilous, while immatures are mostly saprophagous and/or predaceous [33]. In most habitats, especially in northern environments, muscids provide ecological services such as pollination, decomposition, and predation, and they serve as a food source for other vertebrate and invertebrate animals [34-37]. Despite their beneficial ecological contributions, muscid flies are mostly renowned for their medical, veterinary, and agricultural pests, which include the house fly, *Musca domestica* Linnaeus, the stable fly, *Stomoxys calcitrans* (Linnaeus), and various shoot flies of the genus *Atherigona*.

For Muscidae, as for many other Diptera, adult identification is based mostly on chaetotaxy, wing venation, and genitalic structures [31,38]. Their morphology-based identification is often difficult, especially for non-experts, and frequently requires time-consuming genitalic dissections. The identification of adult Muscidae is further complicated by sexual dimorphism as well as by a lack of diagnostic morphological features to differentiate females of some species. The problematic association of conspecific specimens belonging to different genders may, in turn, be further exacerbated by the fact that some species are only described for one sex (mostly male). Despite these complexities, Muscidae have been extensively studied taxonomically for an insect group, especially in northern and alpine habitats of the Holarctic region [38-43]. Consequently, species-level keys (see methods for references) as well as extensive reference collections are available for most Nearctic genera, making this an ideal group for creating a calibration dataset for investigating barcode/morphology correspondence.

The family Muscidae has been very little studied from the perspective of DNA barcoding. The few published studies involving the COI gene in muscid flies have used sequence data to perform phylogenetic analyses [44-47], compare haplotype diversity between populations [48-50], and identify necrophagous species in forensic entomology [51]. Unfortunately, these studies generally focused on COI fragments other than the standard region used for animal DNA barcoding [5,18]. Moreover, all of these studies included only a few species, each often represented by one individual, preventing the rigorous assessment of species limits for closely related taxa and the calculation of intraspecific distances. Muscidae should therefore be targeted for DNA barcoding study to further our understanding of prospects for barcode-based identification of Diptera. This could assist with biodiversity surveys of this important group, especially given challenges such as specimens being damaged using routine collecting techniques (e.g. Malaise and pan traps), important time investments being required for genitalic dissections, as well as the difficulty of identifying females belonging to some closely related species [52].

This study represents the first large-scale barcoding study of the family Muscidae and has three main goals. The first is to use morphologically identified specimens to characterize the range of intraspecific and interspecific divergence values in this family, based primarily upon material from Churchill, Manitoba and other northern localities. Anomalous divergence values (i.e. high intraspecific and low interspecific) are used as notice to re-evaluate the likely “true” species boundaries, using an iterative process including morphological, genetic, and biogeographic information. This contributes to our understanding of the nature of species boundaries and covariation in character types in muscid species. The second goal is to evaluate the degree of correspondence between muscid morphospecies and two different definitions of barcode groupings: a) a clustering and bootstrap-based approach and b) a threshold-based identification method. This work will provide insights useful for the development of identification and biodiversity assessment tools. Thirdly, we use the extensive reference library of DNA barcodes generated in this work, and its accompanying information pertaining to intraspecific and interspecific distances, to address taxonomic problems in the Muscidae, such as cryptic or polymorphic taxa, anatomical variations and male–female associations. Finally, we also contribute to a deeper understanding of the composition of a very rich boreal/arctic transitional fauna, which is investigated in a large Barcoding Biotas biodiversity survey [10].

## Methods

### Specimen selection

A total of 1303 determined Muscidae specimens were selected for sequencing of the Folmer region of COI (Additional file 1). Most (1079) were collected in Churchill, Manitoba, Canada, but 199 and 25 specimens were collected from various Nearctic and Palaearctic localities, respectively (Additional file 1), and added to the Churchill data set to increase the number of individuals belonging to rare or problematic species and to investigate whether the addition of material from geographically distinct populations would increase levels of intraspecific variation. A minimum of two males and two females of each species were included whenever possible, and more specimens were included for variable or ambiguous taxa.

Our analysis was focused on studying the correspondence between morphospecies and barcode groupings for well-characterized species, thus creating a calibration dataset that will be useful for developing identification tools for the muscids and other northern terrestrial Diptera. All specimens were determined to named species or numbered morphospecies (numbers consistent with [52]) prior to sequencing with the exception of “ambiguous” females belonging to six species pairs where they cannot be morphologically distinguished (see [52] for details) and 19 specimens belonging to *Graphomya* Robineau-Desvoidy. Following a preliminary study of most type material of Nearctic *Graphomya*, various errors and inconsistencies found in the work of Artnfield [53] led us to question the validity of most Nearctic *Graphomya* species limits as currently established. Consequently, specimens of *Graphomya* were determined no further than generic level and barcoded as a first step towards a future revision of the genus but excluded from all analyses of species limits. The following reference works were used in specimen identification and to ensure up-to-date taxonomic nomenclature: [31,38-43,54-70]. Species identity for most taxa was then verified through comparison with determined material housed in the Canadian National Collection of Insects, Arachnids and Nematodes, Ottawa, Ontario (CNC); the Bishop`s University Insect Collection, Sherbrooke, Québec, Canada (BUIC); the American Museum of Natural History, New York, NY, USA (AMNH); and the National Museum of Natural History, Smithsonian Institution, Washington, DC, USA (USNM). Voucher specimens are deposited in the BUIC; the CNC; the J.B. Wallis/R.E Roughley Museum of Entomology, University of Manitoba, Winnipeg, Manitoba, Canada (JBWM); and the Biodiversity Institute of Ontario, University of Guelph, Ontario, Canada (BIOUG) (voucher accession numbers (Sample ID) available in Additional file 1).

### DNA barcoding and alignment

Leg-tissue samples consisting of one (occasionally two for small-bodied specimens) legs were removed from specimens and deposited in 96-well plates prefilled with 30 μl of 95% ethanol. All instruments used to remove leg tissues were cleaned in 70% ethanol and sterilized by flame between each specimen. DNA was extracted from tissue samples following standard protocols [71,72]. The barcode region of COI was amplified using LepF1/LepR1 primers; when these primers failed to amplify full-length sequences, the following alternatives were used: LCO1490_t1/HCO2198_t1, LepF1/C_ANTMR1D, MLepF1/HCO2198_t1, MLepF1/LepR1, LepF1/MLepR1 (see Additional file 2 for primer details and references). PCR amplification, product checking, PCR cycle sequencing, and sequencing followed standard protocols employed at the Canadian Centre for DNA Barcoding [73,74]. All specimen collection data, photographs, sequences, PCR and sequencing primers, and trace files are available through the Barcode of Life Data Systems, BOLD [75] under project names: Muscidae (Diptera) of Churchill (MB) and other regions [MCADD], Muscidae (Diptera) of Churchill (MB) and other regions—additional materials [MFDC], and Muscidae and Fannidae of the Aleutian Islands [MFAI] (see Additional file 1 for GenBank accession numbers).

Only high-quality sequences of at least 600 bp and containing less than 1% missing nucleotides (Ns) were retained for data analysis to reduce intraspecific variations due to sequence length [75]. Sequences were translated using the invertebrate mitochondrial code and manually aligned in Mesquite version 2.73 [76]. The alignment was subsequently uploaded to BOLD and MEGA version 5 [77] for data analysis.

### Data analysis

Mean frequencies (%) of each nucleotide and pairs of nucleotide (A + T and C + G) were calculated in MEGA to evaluate whether nucleotide frequencies were comparable to those typical of insects in general for this gene region.

A Neighbor-joining (NJ) tree [78], shown to be a useful clustering method for large datasets [79,80], was built in MEGA for the initial data set using the following parameters of BOLD: Kimura 2-Parameter (K2P) distance model [81] with pairwise deletion of gaps/missing data and inclusion of all substitutions (transitions and transversions). These parameters are recommended by [82] when missing data or gaps are not distributed evenly among aligned sequences as in the case of this data set. K2P distances have been most commonly used in the barcoding literature and were employed to facilitate comparison across studies; while several recent papers have advocated using p-distances instead, results using p-distances vs. K2P are nearly identical [83,84]. Individual node support was assessed by bootstrapping with 1000 replicates [85] (support considered high for values of 95% and higher), and cluster monophyly was assessed to determine the position of females with ambiguous determination and to test the performance of COI in the recovery of morphological species limits. This monophyly requirement is considered to be a strict test of correspondence levels between morphospecies and barcode groupings, as there are mechanisms that can result in species paraphyly [86]. Genetic distances based on the same parameters as those used for building the NJ tree were computed in BOLD and confirmed in MEGA for all specimens excluding *Graphomya*.

As the efficacy of DNA barcoding to discriminate between species and flag potential new species is enhanced if the chosen marker displays levels of intraspecific variability that are lower than the minimum distance to its closest relative, maximum intraspecific distances were plotted against minimum interspecific distances for species with two or more individuals. Specimens of all taxa with maximum intraspecific distance > minimum interspecific distances were reassessed morphologically to investigate potential identification mistakes, undescribed but morphologically distinct lineages, and polymorphic species. Following the recommendations of [87], specimens of all species with more than 2% intraspecific distance were also reassessed to investigate whether they were morphologically homogeneous, and their cluster structure and bootstrap values were examined to identify cases of genetically different but morphologically homogeneous lineages that could represent cryptic taxa (as in [10]). Finally, all specimens belonging to taxa with less than 2% interspecific distance were also re-examined to determine the nature of morphological differences used to distinguish them (e.g. colour characters prone to intraspecific geographical variations or structural features such as genitalia).

Following the morphological reassessment of specimens belonging to the categories described above, decisions were made as to their taxonomic status. Specimen determinations were adjusted accordingly, all genetic distances were recalculated, and the number of haplotypes per species was determined using the DNA barcoding tools available at http://www.ibarcode.org[88]. A new NJ tree reflecting the taxonomic reassessment was built in MEGA with the graphic output showing taxa (instead of individuals), the number of haplotypes per taxon, and the number of sequences for each haplotype. For all species represented by at least two individuals, clustering pattern (species monophyly, paraphyly, or polyphyly) and bootstrap values were examined to assess prospects for identifying Muscidae based on clustering-based approaches. Error rates based on using thresholds alone to classify intraspecific vs. interspecific divergences were assessed in intervals of 0.1%, ranging from 0.1% to 3.0% (approach similar to [19]). Cases of “erroneous lumping” refer to distinct morphospecies that are joined together into a single one using a given threshold, whereas “erroneous splitting” refers to single morphospecies that are divided into two or more taxa at that threshold. The best threshold is characterized as the value minimizing the total number of errors, at the species level.

To determine if the addition of specimens from localities other than Churchill had an influence on intraspecific distances, maximum intraspecific distances were calculated with and without specimens from other regions and compared using randomized permutations in PERM [89] (permutations = 1000, iterations = 10) for all species with material from at least two localities and represented by 2 or more specimens from Churchill. The influence of the number of sequences on maximum intraspecific distances was assessed based exclusively on material from a single region (Churchill) using linear regressions performed in Excel [90].

## Results

Sequencing was successful for 1171 of the 1303 specimens selected for molecular analysis; none of these had more than 1% missing nucleotides, but 38 were less than 600 bp long and therefore excluded, a procedure that did not eliminate any taxa from our data set (Additional file 1). When excluding 19 sequences from *Graphomya* spp., our data set contained 1114 high-quality sequences (951 from Churchill) representing 160 species from 25 genera (Additional files 1, 3), and included 89% of the known fauna from Churchill [52].

Inspection of the final alignment revealed no stop codons, insertions, or deletions. Mean nucleotide content of COI sequences was: A (30.0%), T (39.3%), C (15.4%), and G (15.4 %). As reported for some Muscoidea [46] and other dipteran mitochondrial sequences [5,27], A + T (69.2%) was in higher proportion than C + G (30.8%).

Ten of the 12 taxa with ambiguous females formed distinct clusters on the NJ tree (Additional file 3), therefore allowing for the determination of females belonging to the following species pairs via genetic matching with the identified males: *Coenosia tarsata* Huckett and *C. verralli* Collin, *Limnophora rotundata* Collin and *Limnophora* sp. 2, *Phaonia consobrina* (Zetterstedt) and *P. rugia* (Walker), *Schoenomyza dorsalis* Loew and *S. litorella* (Fallén), and *Spilogona atrisquamula* Hennig and *S. pusilla* Huckett. Specimens of the remaining pair, composed of *Thricops septentrionalis* (Stein) and *T. spiniger* (Stein), formed a single mixed cluster and shared some identical haplotypes (Additional file 3). The examination of the remaining clusters on the NJ tree (Additional file 3) revealed paraphyly and polyphyly issues involving six additional taxa (Additional file 3, Table 1). Overall, congruence between morphology (initial determinations) and molecular species limits, based on cluster monophyly with high (≥95%) bootstrap support, was found in 128 of the 136 morphologically-defined taxa (94.1%) represented by 2+ individuals; *Spilogona atrisquamula* and *Coenosia comita* individuals also clustered together with conspecifics but with lower bootstrap support (53% and 93% respectively) (Additional file 3).

**Table 1:** Details of taxonomic reassessment

**Taxon**	**CO1 distances (%)**				**Outcome of taxonomic reassessment**
	**Pre**		**Post**		
	**max intra**	**min inter**	**max intra**	**min inter**	
*Coenosia demoralis*	—	1.47	—	1.47	Morphologically distinct
*Coenosia minor*	0.17	1.47	0.17	1.47	Morphologically distinct
*Drymeia pribilofensis*	0.15	1.38	0.15	1.38	Morphologically distinct
*Drymeia segnis*	0.00	1.38	0.00	1.38	Morphologically distinct
*Helina evecta*	3.01	4.55	3.01	4.55	Morphologically distinct
*Helina laxifrons*	2.54	5.72	2.54	5.72	Morphologically distinct
*Hydrotaea pilitibia*	0.00	1.47	0.00	1.47	Morphologically distinct
*Hydrotaea scambus*	—	1.47	—	1.47	Morphologically distinct
*Lispe cotidiana*	0.00	1.54	0.00	1.54	Morphologically distinct
*Lispe uliginosa*	0.00	1.54	0.00	1.54	Morphologically distinct
*Lispocephala varians*	—	0.48	X	X	Misidentification of *L. erythrocera*
*Lispocephala erythrocera*	0.00	0.48	0.65	6.74	Min inter >2% after misidentification resolution
*Muscina flukei*	0.00	1.86	0.00	1.86	Morphologically distinct
*Muscina levida*	4.20	1.86	0.17	3.80	Problematic specimen renamed *Muscina* sp.
*Phaonia errans*	4.24	7.95	0.80	3.27	Split into *P. errans* and *Phaonia luteva*
*Phaonia savonoskii*	0.16	1.70	0.16	1.70	Morphologically distinct
*Phaonia serva*	0.46	1.70	0.46	1.70	Morphologically distinct
*Spilogona arctica*	0.61	1.75	0.61	1.75	Morphologically distinct
*Spilogona atrisquamula*	2.50	2.01	2.50	2.01	Morphologically distinct
*Spilogona contractifrons*	3.80	1.23	0.80	1.75	Split into *S. contractifrons* and *Spilogona* sp. 12
*Spilogona fatima*	0.00	1.70	0.00	1.70	Morphologically distinct
*Spilogona forticula*	0.00	1.23	0.00	1.23	Morphologically distinct
*Spilogona novemaculata*	0.00	1.70	0.00	1.70	Morphologically distinct
*Spilogona* sp. 8	—	0.00	X	X	misidentification of *Spilogona* sp. 1
*Spilogona* sp. 1	0.00	0.00	0.00	4.35	Min inter >2% after misidentification resolution
*Thricops hirtulus*	0.15	1.70	0.15	1.70	Morphologically distinct
*Thricops innocuus*	0.61	1.70	0.61	1.70	Morphologically distinct
*Thricops spiniger*	0.77	0.00	X	X	Lumped with *Thricops septentrionalis*
*Thricops septentrionalis*	0.93	0.00	0.93	0.77	Renamed *Thricops septentrionalis/ spiniger*
*Thricops villicrus*	0.15	0.77	0.15	0.77	Morphologically distinct

Outcome of the taxonomic reassessment for all 30 Muscidae taxa with monophyly issues and/or anomalous divergence values in the pre-assessment data set. Maximum pairwise intraspecific (max intra) and minimum pairwise interspecific (min inter) distances shown for pre-assessment (Pre) and post-reassessment (Post) data sets. Missing intraspecific distances in taxa represented by a single sequence denoted by ---, missing distance values caused by the absence of a taxon in the post-reassessment data set denoted by X.

**Figure 1:**
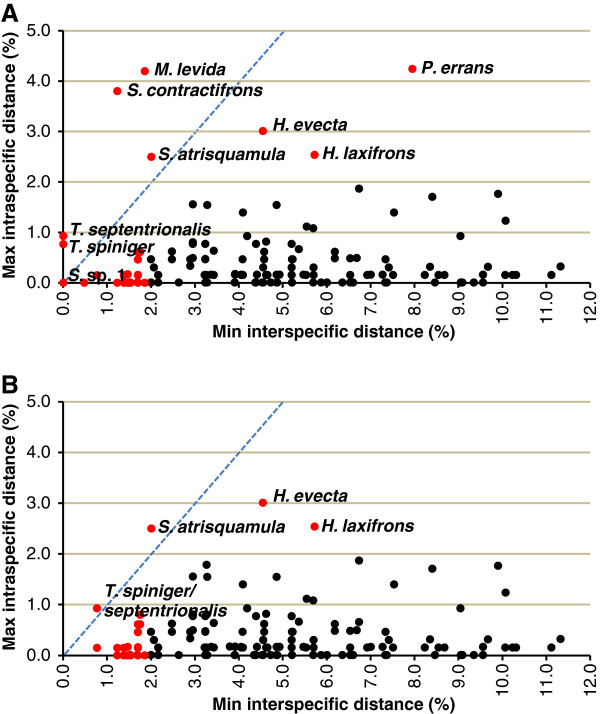
**COI distances of muscid taxa in pre (A) vs post (B) reassessment data sets.** Minimum interspecific distance plotted against maximum intraspecific distance for all morphologically defined taxa represented by 2 or more individuals (n=136 in (**A**); n=137 in (**B**)). Red data points indicate “anomalous” divergence values (see text for details). Named data points indicate maximum intraspecific distance >= minimum interspecific distance and/or maximum intraspecific distance > 2.0%. Stippled blue diagonal marks the line of equal values for intra and interspecific distances.

Using our initial morphological identifications, pairwise intraspecific distances calculated for the 136 taxa represented by two or more individuals ranged between 0 and 4.24% (average of means = 0.22%; average of maxima = 0.48%). Minimum interspecific distances to nearest neighbour for all 160 taxa ranged from 0 to 11.33% (average = 4.72%). Anomalous divergence values were found in 30 taxa (Table 1, Figure 1A).

### Taxonomic reassessment

**Figure 2:**
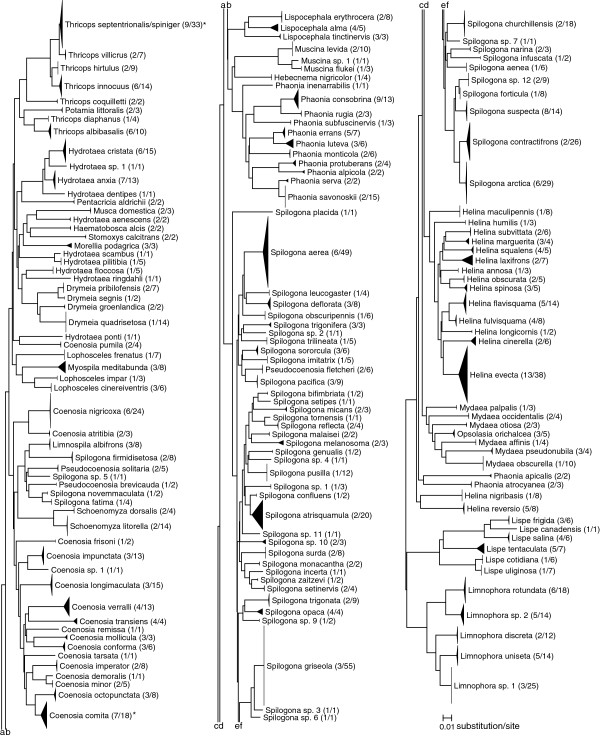
**Neighbour-joining tree of post-reassessment data set.** Kimura 2-parameter NJ tree representing 1114 COI sequences from 160 Muscidae species. For each taxon terminal, height of the triangle represents the number of specimens and width represents the extent of intraspecific divergence. Species with bootstrap values below 95% indicated with an asterisk. Numbers in parentheses represent the number of haplotypes and the number of sequences for each taxon.

The taxonomic reassessment of 30 of the 160 species in the data set resulted in changes in the limits and/or genetic distances of 9 taxa, including all those with non-monophyly issues, while the remaining 21 were morphologically homogeneous (Table 1). Two cases of misidentifications were discovered; the specimen originally identified as *Lispocephala varians* Malloch belongs in fact to *L. erythrocera* (R.-D.), and *Spilogona* sp. 8 was found to be identical to *Spilogona* sp. 1 and therefore renamed accordingly. We also uncovered three cases of morphologically distinct lineages originally assigned to the same taxon. The highest intraspecific divergence value was found in *Phaonia errans* (Meigen) (Figure 1A) and upon re-examination, the two internal clusters of *P. errans* (Additional file 3) were renamed *P. errans* and *Phaonia luteva* (Walker) **stat. nov.** (Figure 2), as material from each barcode cluster corresponded to a distinct Nearctic subspecies or variety of *P. errans* recognized by various authors [60,64]; but see [38] for synonymy details. We found consistent differences in external and male genitalic characters between specimens belonging to the two clusters of *S. contractifrons* (Zetterstedt) (Additional file 3), which were not sisters, one corresponding to the nominal species, and the second renamed *Spilogona* sp. 12, as it did not correspond to any known Nearctic or Palearctic species (Table 1, Additional file 1). A similar situation involves *Muscina levida* (Harris), where a number of differences were found in the single genetically divergent specimen (Additional file 3), which was consequently renamed *Muscina* sp. 1 (Table 1, Additional file 1).

The last reassessment issue concerns the mixed cluster composed of *T*. *septentrionalis* and *T*. *spiniger* (Additional file 3). While there are consistent external morphological differences between males (fore tibia with 4–5 posteroventral spines and mid tarsomere 4 no longer than wide in *T. septentrionalis*; fore tibia with 3 spines and mid tarsomere twice as long as wide in *T. spiniger*), there are no genitalic differences between them, and the females cannot be separated [41]. Therefore, our results indicate that either COI does not discriminate between these two species, or that males of the group express two distinct morphs. Since further testing of species limits in these taxa is beyond the scope of the present work, we pooled all specimens belonging to these taxa together under the name *T*. *septentrionalis*/*spiniger* to recalculate distance measures but indicate a taxonomic issue left to be resolved (Table 1, Additional file 1). All changes were implemented in the post-reassessment NJ tree (Figure 2).

**Figure 3:**
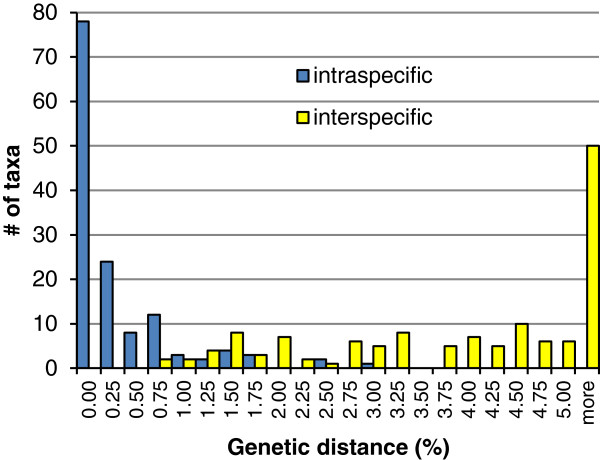
**Frequency distribution of intra and interspecific distances.** Distribution of maximum intraspecific and minimum interspecific pairwise distances in intervals of 0.25% for 137 Muscidae species represented by 2 or more individuals in the post-reassessment data set.

Identity changes resulting from the taxonomic reassessment did not modify the total number of taxa in the post-reassessment data set, as three species were split while three other pairs were lumped, but it brought the number of taxa represented by 2 or more specimens to a total of 137. Pairwise intraspecific distances in the post-reassessment data set ranged from 0 to 3.01% (average of means = 0.19%; average of maxima = 0.42%), whereas minimum interspecific distances to nearest neighbour ranged from 0.77 to 11.33% (average = 4.82%) (Figures 1B and 3). Twenty-two taxa (19 represented by 2 or more specimens) exhibited less than 2% interspecific distance to their nearest neighbour (Figures 1B and 3). Maximum intraspecific distance was slightly higher than minimum interspecific distance in only two taxa, *T*. *septentrionalis*/*spiniger* and *S. atrisquamula*, and this last species, along with *Helina evecta* (Harris) and *Helina laxifrons* (Zetterstedt), were the only three with an intraspecific distance greater than 2% (Table 1, Figure 1B). The taxonomic reassessment also confirmed that the 16 distinct morphospecies that could not be associated with valid names were genetically distinct from all named taxa (Figure 2, Additional file 1).

#### Clustering vs. thresholds

**Figure 4:**
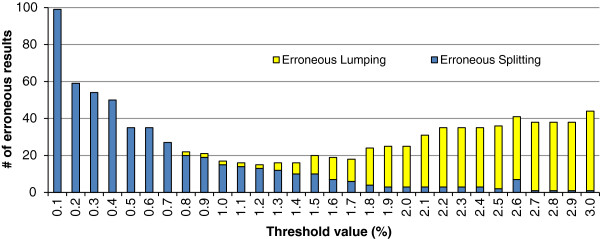
**Cumulative error rates per pairwise distance threshold value.** Hypothetical threshold values were evaluated in intervals of 0.1% pairwise distance values to assess how well they performed in separating intraspecific from interspecific divergence values, assuming that the morphospecies are true representations of species boundaries. Observed genetic distances were calculated using the post-reassessment dataset. At a given test threshold value, “erroneous lumpings” refer to cases of distinct morphospecies that are grouped together into one provisional species, due to having nearest neighbour interspecific genetic distances that fall below the threshold. “Erroneous splittings” refer to single morphospecies split into two or more provisional species at that threshold, due to having a maximum intraspecific divergence above the threshold.

Using a clustering-based method, all individuals of 134 of 137 species (97.8%) represented by 2+ individuals (post-reassessment determinations) grouped together with their conspecifics with high (≥95%) bootstrap support. The three remaining taxa also grouped with conspecifics, but two with ≥50% bootstrap support and the *T. septentrionalis/spiniger* cluster with 27%, further emphasizing a taxonomic issue left to be resolved (Figure 2). By contrast, threshold-based methods would yield a maximum species-discrimination success rate of 90%, at a threshold of 1.2% (Figure 4). Due to the balance of false lumping and false splitting being more even at a higher threshold, a 1.5% threshold would yield the highest accuracy in estimating species richness.

The inclusion of 94 sequences from localities other than Churchill did not significantly alter the maximum intraspecific distance (one-tail permutation test: *P* = 0.09) of the 28 taxa to which they belong. Maximum intraspecific distance in the 119 taxa represented by at least 2 specimens in Churchill, for a total of 924 specimens, was positively correlated with the number of sequences per taxon (R^2^ = 0.07, *P* = 0.002).

## Discussion

The performance of DNA-based specimen identification in Diptera using COI varies greatly in the literature. Identification success, when using a monophyly criterion, ranges from less than 50% in one genus of Calliphoridae [91] to over 90% in most other families studied [12,13,26,92]. We show that DNA barcoding is a highly efficient tool for the identification of northern Nearctic muscid flies, as we report congruence levels of 98% between morphological and molecular species limits in 160 taxa when using a clustering approach and enforcing strict monophyly and high bootstrap requirements. This value rises above 99% upon relaxing the bootstrap requirement; just one case of a mixed cluster of two species remained in our dataset following post-barcoding morphological reassessment, representing a single conspicuous taxonomic puzzle.

### Characterization of genetic divergence

In one of the first attempts to characterize levels of genetic divergence among congeneric species across various taxa [18], it was determined that a threshold of 2% generally separated levels of intra and interspecific sequence divergence in most invertebrate taxa. It has since been demonstrated that levels of intra and interspecific variation will generally partially overlap in well-populated data sets [19]. Both the range and the average of genetic divergences detected will vary according to the taxonomic group selected and be influenced by the phylogenetic relatedness of selected species, as well as by the number and geographical distribution of species and specimens in a data set [19,28,29,93,94]. In general, intraspecific divergences are expected to increase and interspecific divergences to decrease with more comprehensive taxonomic sampling [19], larger geographic scope [28], and the inclusion of more stable environments, such as tropical lowlands [29], where extinction rates are expected to be lower. Despite these considerations, datasets often show that DNA barcodes retain the ability to discriminate species—and to elucidate undescribed diversity—even across large geographic regions [93,95] and in rich tropical insect faunas [7,12-14,96]; but see [29].

In Diptera, ranges of 0.17-1.20% and 3.00-5.40% have been reported for average of the means and maxima of COI intraspecific distances, respectively [12,26,27,92,97]. The values reported here for our post-reassessment Muscidae data set are comparable yet at the lower end of these ranges (average of the means 0.18%; maximum of 3.01%). The constrained intraspecific divergences here may reflect several factors, such as the high quality of the prior species-level taxonomic work in the Muscidae, our having conducted genitalic examination of most specimens, as well as the northern geographic focus of our work. The relative completeness of the taxonomy of the northern Muscidae is affirmed by the fact that only a small proportion of genetic clusters in our study, which were also separated from relatives by morphological characters, could not be linked with named species (16 of 160 = 10%). Despite these likely explanations for our comparatively low intraspecific divergences, it is challenging to interpret differences in levels of intraspecific genetic divergence among taxa for which different character sets are used for taxonomy. We suggest that the near-complete correspondence between genetic groupings and morphospecies for the Muscidae gives added weight both to DNA barcodes and to the morphological characters typically used for species-level diagnosis in Muscidae taxonomy (mainly chaetotaxy and genitalia). The correspondences suggest that both are likely to be revealing the true underlying species boundaries, which remain unknown to us.

Several additional factors beyond taxonomy, such as the number of sequences or the inclusion of sequences from a range of geographic localities, can influence the extent of genetic divergences measured within species [19,28,98]. Despite theoretical concerns that intraspecific divergences will increase dramatically when studies are conducted at large spatial scales, the majority of empirical evidence to date indicates that this is a more modest problem for DNA barcoding than originally envisaged. Bearing in mind that only 28 species could be included in our analysis, the inclusion of sequences from localities other than Churchill did not have an influence on maximum intraspecific distance in our dataset. These results are comparable to those of Hebert *et al*. [95], who reported low intraspecific variation among 11,289 sequences of lepidopteran species (1327 species in 62 families) collected from different localities in eastern North America, as well as the results of Lukhtanov *et al.*[93] for Central Asian butterflies. By contrast, the Trichoptera (caddisflies) of North America [99] as well as diving beetles (tribe Agabini) of the western Palearctic [98], which both inhabit freshwaters expected to be more divided than terrestrial insect habitats, exhibit increasing intraspecific genetic divergence at large spatial scales. Part of this increase may be attributable to previously unrecognized species being lumped together under current names; despite this issue, DNA barcoding remained effective (90-93%) at distinguishing named morphospecies within these taxa at continental spatial scales [98,99]. It appears, then, that global sequence libraries of insects may serve as references for local species identification for newly studied sites, at least for many groups in the temperate and polar zones. Success rates are particularly high for vagile groups (such as Lepidoptera), while even for more challenging groups identification success can be near 100% at smaller spatial scales or when employing joint geographic and genetic data [98]. Further work on the question of barcode variability at very large spatial scales is particularly required in tropical environments, as the majority of tropical insect DNA barcoding studies to date have included a relatively modest regional spatial scale (e.g. [7,8,12-14,29,100].

As with intraspecific distance values reported here, the minimum (0.77%) and average (4.82%) of the nearest neighbour interspecific distances for the post-reassessment data set were lower than most interspecific distances found in the literature for insects, including mosquitoes [26], black flies [27], bees [6], mayflies, stoneflies and caddisflies [10], and springtails [101], but comparable to those reported for tachinid flies [12]. However, some studies report average congeneric divergences rather than nearest-neighbour distances as employed here, which provide the more stringent test of discriminating the closest relatives [24]. In their foundational work, Hebert *et al*. [18] reported that more than 98% of invertebrate taxa they investigated (including 177 species of Diptera, but no Muscidae) showed more than 2% pairwise distance to their nearest neighbour. In contrast, only 86% of the 160 taxa in the present work were separated from their nearest neighbour by a distance greater than 2%. This difference is attributable to our focus on numerous species from a single family (89% of the fauna of Churchill [52]), and approximately half of the arctic and subarctic Nearctic fauna [38], as opposed to the taxonomically broad but poorly populated data set of Hebert *et al*. [18]. Limits of species with distance to nearest neighbour < 2% in our data set were supported by morphological characters, but these were occasionally subtle and/or only detectable in the males, possibly suggesting a recent divergence time [23].

As to be expected from a well-populated data set [19,98], we report an important overlap in the range of intra and interspecific distances for our data set, clearly indicating a lack of “barcoding gap” [19] in muscid flies. While distance-based methods for species determination have been extensively criticized (e.g. [19,24]), it was through the combination of cluster examination on the NJ tree and the use of 2% as an arbitrary divergence threshold to identify “anomalous” distance values that we were able to rapidly pinpoint and address taxonomic issues in our original data set, as well as confirm that minimum interspecific distance in Muscidae ranges well below 2% for many species.

It is important to expand upon our above understanding of divergence patterns in the Muscidae by including specimens from warm temperate and tropical regions. The often-low interspecific divergences we found between sibling species present in Churchill were associated with reciprocal monophyly in the vast majority of cases. In more southerly regions, higher richness combined with greater intraspecific genetic structure have been described as presenting a challenge for barcode-based species discrimination [28]. Incomplete lineage sorting among many young species pairs would complicate the clustering-based identification approach advocated here for the northern muscids. However, barcode results to date for some tropical insect faunas are promising (e.g. [12-14,102]; but see [29]).

Supposedly depauperate northern regions might be expected to be an “easy” test for barcoding due to lower species richness and lineage pruning during glaciations, as has been demonstrated for fish, for example [103]. However, our usage of Churchill and other northern regions may, in fact, provide a relatively stringent test of barcoding success for the Muscidae. Being one of the most speciose and broadly distributed family of terrestrial insects in northern regions [32], muscids are likely to have been strongly influenced by glaciations, and our observed shallow interspecific divergences among many pairs of congenerics suggest recent speciation events during the Pliocene and Pleistocene, when applying an approximate molecular clock calibration to our divergences (e.g. [104]). Moreover, the Churchill region is a zone of admixture from Beringian, high arctic, and southerly refugia (e.g. [105]). This combination of factors may lead to mixing of intraspecific lineages from different refugia as well as young species in the Churchill region. Further data from additional geographic regions will be desirable to confirm that the patterns reported here are broadly applicable for all of Muscidae, but we optimistically predict that muscids will be broadly amenable to barcoding.

### Future success rate of barcode-based identification of unknowns

Congruence between morphological and molecular species limits was 97.8% when using a clustering approach with high bootstrap support and enforcing a monophyly requirement in the molecular results, while clustering and identification success was 99% using clustering with a relaxed bootstrap criterion. We found this high level of correspondence to be surprising, given that monophyly is considered a strict test of species limits. Funk and Omland [86] reported that up to 23% of species may be paraphyletic or polyphyletic; however, they noted that this proportion declines in better-studied taxa, suggesting that a portion of this total reflects incomplete taxonomic knowledge.

By contrast, threshold-only based methods would yield lower success for grouping unknown individuals into species units, with a maximum success rate of 90% found at a threshold of 1.2%, which is less than half of the threshold value found to minimize error rate for a group of marine molluscs [19]. While we recommend combining distance and cluster-based approaches for taxonomic and faunistic works concerned with “true” species boundaries and numbers, such a level of success would permit rapid assessments of approximate species richness in unknown faunas. Furthermore, a combination of clustering and threshold-based approaches would allow new taxa or singletons to be flagged as likely new species. Our results also may contribute to the development of relaxed clustering methods, whereby divergences exceeding specified thresholds are permitted. Moreover, our study demonstrates the great utility of having well-populated species-level reference libraries; we have found that neither small interspecific distances nor large intraspecific distances will derail identification success when there are many reference sequences against which to match unknowns.

While specimens of *Graphomya* were excluded from all analyses of species limits due to taxonomic issues, at a threshold of 1.2%, our 19 sequences form five putative species and the two lineages represented by more than one specimen are monophyletic with high bootstrap values (Additional file 3). Since only one of these five putative species contains at least two specimens of the same sex, the barcoding of additional individuals will be necessary before it can be determined if these lineages are all distinct morphologically and if they correspond, at least in part, to the Nearctic species as defined in Arntfield [53].

In contrast to the results obtained at the species level, generic limits were poorly supported by COI in the NJ tree (Figure 2), with more than half of the genera represented by two or more species being para- or polyphyletic. It appears, then, that muscid specimens cannot be reliably identified to genus using COI based solely on association with closely related taxa, at least when based on the NJ method of tree building. The percentage of insect genera forming monophyletic clusters based exclusively on COI varies greatly in the literature, with values similar to those reported here in ithomiine butterflies (50-61% depending on clustering method) [29] and black flies (62.5%) [27], but much higher in bees (100%) [6]. It remains unclear whether this is due to lack of phylogenetic signal in COI at this depth, the type of tree-building method, or to the true lack of monophyly of genera as currently defined; further phylogenetic work involving a multi-gene approach is required to address the prospects for higher-level taxonomic assignments in Diptera based upon COI.

### DNA barcoding and Nearctic Muscidae taxonomy

The DNA barcode reference library produced in our work allowed us to resolve the problematic issue of male/female associations for 5 of our 6 ambiguous species pairs as well as confirm or challenge our diagnosis of sex associations for members of unnamed morphospecies. Our results demonstrate that a well-populated reference library not only facilitates the association of conspecific specimens or the detection of identification errors, but that it also contributes to the taxonomic workflow through discovering morphologically distinct taxa and challenging accepted species limits. The discovery of *Spilogona* sp. 12 was especially significant, as it allowed Jolicoeur and Savage (personal communication) to document that the most abundant species of Schizophora (Diptera) on the alpine tundra of the McGerrigle mountains of the province of Québec is, in fact, the undescribed muscid *Spilogona* sp. 12 rather than the similar *Spilogona contractifrons*, recorded in the literature from the northern Appalachians and numerous other Nearctic localities [38,106]. While we confirm the presence of both *Spilogona* sp. 12 and *S. contractifrons* in Churchill, the Nearctic distribution of the latter will need to be entirely reassessed in light of this new discovery.

The taxonomic reassessment also led to the reinstatement of *Phaonia luteva***stat. nov.** as a species distinct from *P. errans*. Malloch [64] recognized three distinct Nearctic varieties of *Phaonia errans*: a yellow-legged variety, *Phaonia errans errans* (Meigen); a dark-legged variety, *Phaonia errans varipes* (Coquillet); and a variety with rufous-yellow legs and distinctive chaetotaxy, *Phaonia errans completa* Malloch. Huckett [107] synonymized *varipes* Coquillet with *Anthomyia luteva* Walker and treated the dark-legged form as *Phaonia errans* var. *luteva* in later publications [38,60]. Since specimens of *Phaonia errans sensu lato* clustered here into distinct yellow and dark-legged branches separated by more than 4% intraspecific distance (higher than all other taxa in this work), we concluded that the dark-legged specimens belonged to *P. luteva* as interpreted by Huckett [38] based on his examination of Walker’s type [107] and that this taxon should be recognized as a full species distinct from *P. errans*. Specimens of *Phaonia errans* var. *completa* were not available for DNA extraction in the context of this work but the distinctive leg colour and chaetotaxy of this taxon suggest that it might also be a separate species rather than a regional variety of *P. errans*.

A very low level of genetic divergence between species, well below the delineated threshold, may reflect intraspecific polymorphism. Of all the morphologically distinct taxa included in this work, only *T*. *septentrionalis* and *T*. *spiniger* shared identical haplotypes. While males of these taxa can be easily distinguished morphologically (see results section), they share a mostly overlapping Nearctic distribution [38,41]. In a phylogenetic analysis of *Thricops* based on a combination of morphological and nuclear characters including COI, COII, and the nuclear gene *white*, Savage *et al*. [44] treated the two species as distinct but very closely related. Savage *et al*. [44], however, included only one specimen of each taxon in the analysis, therefore preventing an assessment of intraspecific vs interspecific distances. Based mostly on geographical distribution data for these two taxa, we suspect that *T*. *septentrionalis* and *T*. *spiniger* may belong to one polymorphic species. In order to test this hypothesis, and before permanent changes are made to their taxonomic status, the genetic distance between *T*. *septentrionalis* and *T*. *spiniger* should be further assessed with other markers capable of distinguishing between closely related species as done by Whitworth *et al*. [91], who found that COI and COII underestimated species numbers in the genus *Protocalliphora* but that the analysis of amplified fragment length polymorphism (AFLP) generated clusters corresponding to morphological *Protocalliphora* species limits. Mitochondrial DNA introgression associated with *Wolbachia* infection, a factor that has been proposed to explain a lack of correspondence between COI and morphology in insects [91,108], could also possibly explain the presence of shared haplotypes between *T. spiniger* and *T. septentrionalis*. The high congruence between molecular and morphological species limits in our study suggests, however, that mitochondrial DNA introgression is not common in our data set.

An important application of DNA barcoding is the discovery of cryptic species, revealed through large intraspecific divergence values in an otherwise morphologically uniform taxon. In Diptera, cryptic species appear to be especially common in parasitoid flies of the family Tachinidae [12,13], but no information was available for muscid flies prior to this study. In the post-reassessment data set, only *H*. *evecta*, *H. laxifrons* and *S. atrisquamula* demonstrated maximum levels of intraspecific distances greater than 2% (but still no higher than 3.01%) coupled with homogeneous morphological characters. As there is nothing among the scant information currently available on the ecology of these species suggesting the presence of distinct internal lineages [33], we retained the currently accepted species limits for these taxa. However, we recommend the analysis of further molecular data such as the Internal Transcribed Spacers (ITS) region of the ribosomal DNA, a marker that has performed well to confirm the presence of cryptic lineages in the Diptera genera *Belvosia* (Tachinidae) [12] and *Chrysomya* (Calliphoridae) [109].

## Conclusion

The comprehensive and highly detailed morphology-based taxonomic works available for the Muscidae (see Methods for complete list) have allowed us to complete an in-depth assessment of congruence levels between molecular and morphological species limits in northern muscid flies and to evaluate the identification success rates of threshold and cluster-based methods. Our results and the approach undertaken in this study indicate that the iterative process by which specimen identification is revaluated in light of barcoding results improves the robustness of the reference library produced, and that the evaluation of the performances of DNA barcoding as an identification tool is much more accurate when all voucher material (instead of a list of names and sequences downloaded from sequence databases) is available for consultation.

This study provides a DNA barcode reference library for nearly half the northern Nearctic Muscidae and contributes almost all of the vouchered barcode records for this family now available through BOLD. Given our near-comprehensive coverage of the muscid fauna of Churchill, it will now be possible to use DNA barcodes to identify many individuals within this abundant and ecologically important family within Churchill and other northern regions. This will open new avenues for research into subjects such as larval ecology, male/female phenology [110], and habitat associations. Combined with other studies being conducted in collaboration with the Churchill “Barcoding Biotas” campaign ( [10]), there will also be new opportunities for research into species interactions, community ecology, and large-scale faunal shifts linked to climate change. These possibilities demonstrate the value of detailed studies of focal taxa combined with the shared vision of using standardized markers and focal geographic regions to elucidate biodiversity.

## Misc

Anaïs K Renaud and Jade Savage contributed equally to this work.

## Competing interests

The authors declare that they have no competing interests.

## Authors’ contributions

AKR conducted the bulk of specimen preparation and identification, participated in the study design, sequence alignment, data analysis, and drafted parts of the manuscript. JS participated in specimen identification, contributed to the conception, design and coordination of the study, participated in data analysis, and helped drafting the manuscript. SJA contributed to the study design, sequence alignment and data analysis, and helped to draft the manuscript. All authors read and approved the final manuscript.

## Authors’ information

AKR completed a MSc degree from the University of Manitoba in 2012 and is currently working for a non-profit organization involved with wetland conservation in south eastern Québec. JS is an Associate Professor in the Department of Biological Sciences of Bishop’s University and the Director of the Bishop’s University Insect Collection; she is interested in the systematics and biodiversity of muscoid Diptera. SJA is an Assistant Professor in the Biodiversity Institute of Ontario & Department of Integrative Biology of the University of Guelph and is interested in evolutionary community structure, macroevolution, and using genetic tools to elucidate biodiversity.

## Supplementary Material

Additional file 1 **Specimen list.** Name (pre and post-reassessment), Sample ID (voucher accession number), GenBank Accession Number, Process ID, Specimen repository and collection locality of 1303 specimens submitted for DNA amplification. Post-reassessment name provided only for specimens that yielded high quality sequences of at least 600 base pairs. Blue text indicates a successful amplification but low quality sequence (<600bp); red text indicates a failure to amplify; bolded text indicates a name change following the taxonomic reassessment. Click here for file

Additional file 2 **List of primers.** Forward and reverse primers used to amplify COI sequences of muscid flies. The specific primers used for PCR and sequencing of each specimen are available through BOLD ( http://www.boldsystems.org). Click here for file

Additional file 3 **Neighbour-joining tree of pre-reassessment data set.** Kimura 2-parameter NJ tree of 1133 high quality COI sequences (≥600bp) from 160 Muscidae species and undetermined material of *Graphomya*. The five putative lineages of *Graphomya* based on 1.2% threshold are highlighted in different colours. Bootstrap values based on 1000 replicates. Click here for file
