# Supplementary material for: DNA barcoding of Northern Nearctic Muscidae (Diptera) reveals high correspondence between morphological and molecular species limits
Source: BMC Ecol. 2012 Nov 23;12:24. doi: 10.1186/1472-6785-12-24 (PMC3537539; doi:10.1186/1472-6785-12-24)
Supplement: Additional file 2 — List of primers. Forward and reverse primers used to amplify COI sequences of muscid flies. The specific primers used for PCR and sequencing of each specimen are available through BOLD ( http://www.boldsystems.org). [file 1472-6785-12-24-S2.docx]

### Additional file 2 – List of primers

Forward (F) and reverse (R) primers used to amplify COI sequences of muscid flies. The specific primers used for PCR and sequencing are available for all specimens through BOLD ([www.boldsystems.org](http://www.boldsystems.org)). Unless otherwise specified in footnotes, the same primers are used for PCR amplification and cycle sequencing.

| **Primer pair** | **Sequence (5’ to 3’)** | | **References^a^ F/R** |
| --- | --- | --- | --- |
|  | **F** | **R** |  |
| LepF1/LepR1 | ATTCAACCAATCATAAAGATATTGG | TAAACTTCTGGATGTCCAAAAAATCA | 1/1 |
| LCO1490_t1/HCO2198_t1 | TGTAAAACGACGGCCAGTGGTCAACAAATCATAAAGATATTGG | CAGGAAACAGCTATGACTAAACTTCAGGGTGACCAAAAAATCA | 2&3/2&3^b^ |
| LepF1/C_ANTMR1D | ATTCAACCAATCATAAAGATATTGG | (Cocktail)^c^ | 1/4 |
| MLepF1/HCO2198_t1 | GCTTTCCCACGAATAAATAATA | CAGGAAACAGCTATGACTAAACTTCAGGTGACCAAAAAATCA | 5/2&3^b^ |
| MLepF1/LepR1 | GCTTTCCCACGAATAAATAATA | TAAACTTCTGGATGTCCAAAAAATCA | 5/1 |
| LepF1/MLepR1 | ATTCAACCAATCATAAAGATATTGG | GCTTTCCCACGAATAAATAATA | 1/5 |

^a^ [1] Hebert PDN, Penton EH, Burns J, Janzen DH, Hallwachs W: **Ten species in one: DNA barcoding reveals cryptic species in neotropical skipper butterfly, *Astraptes fulgerator.*** *PNAS* 2004, **101:**14812-14817; [2] Folmer O, Black M, Hoeh W, Lutz R, Vrijenhoek R: **DNA primers for amplification of mitochondrial cytrochrome c oxidase subunit I from diverse metazoan invertebrates.** *Mol Mar Biol Biotechnol* 1994, **3:**294-299; [3] Messing J: **New M13 vectors for cloning**. *Method Enzymol* 1983, **101**:29–71; [4] Smith MA, Rodriguez JJ, Whitfield JB, Deans AR, Janzen DH, Hallwachs W, Hebert PDN: **Extreme diversity of tropical parasitoid wasps exposed by iterative integration of natural history, DNA barcoding, morphology, and collections.**  *PNAS* 2008, **105:**12359-12364; [5] Hajibabaei M, Janzen S, Burns JM, Hallwachs W, Hebert PDN: **DNA barcodes distinguish species of tropical Lepidoptera.** *PNAS* 2006, **103:**968-971; [6] Simon C, Frati F, Beckenbach A, Crespi B, Liu H, Flook P: **Evolution, Weighting, and Phylogenetic Utility of Mitochondrial Gene-Sequences and a Compilation of Conserved Polymerase Chain-Reaction Primers**. *Ann Entomol Soc Am* 1994, **87:**651-701; [7] Smith MA, Fisher BL, Hebert PDN: **DNA Barcoding for effective biodiversity assessment of a hyperdiverse arthropod group: the ants of Madagascar**. *Philos Trans R Soc Lond B Biol Sci*. 2005, **360:**1825-1834.

^b^ The full “tailed Folmer” sequences are used for PCR amplification [2,3], while the M13 tail sequences [3] are used for cycle sequencing (M13F-TGTAAAACGACGGCCAGT; M13R-CAGGAAACAGCTATGAC).

^c^ C_ANTMR1D is a cocktail of two overlapping and degenerate primers that, when used in combination with LepF1, produces an amplicon in insects that ranges in size from 290-300 bp [4]. Only the LepF1 primer is used for sequencing. The two constituent sequences and original references are:

C_ANTMR1D-RonIIdeg_R: GGRGGRTARAYAGTTCATCCWGTWCC [Modified [6]]

C_ANTMR1D-AMR1deg_R: CAWCCWGTWCCKRMNCCWKCAT [Modified from [7]]
