# Supplementary material for: DNA barcoding of Northern Nearctic Muscidae (Diptera) reveals high correspondence between morphological and molecular species limits
Source: BMC Ecol. 2012 Nov 23;12:24. doi: 10.1186/1472-6785-12-24 (PMC3537539; doi:10.1186/1472-6785-12-24)

Additional file 3 – Neighbour-joining tree of pre-reassessment data set.

Kimura 2-parameter NJ tree of 1133 high quality COI sequences (≥600bp) from 160 Muscidae species and undetermined material of *Graphomya*. The five putative lineages of *Graphomya* based on 1.2% threshold are highlighted in different colours. Bootstrap values based on 1000 replicates.

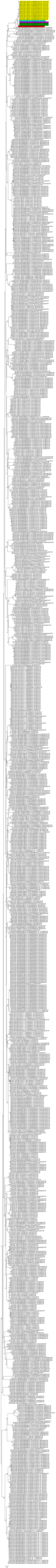

Supplement: Additional file 3 — Neighbour-joining tree of pre-reassessment data set. Kimura 2-parameter NJ tree of 1133 high quality COI sequences (≥600bp) from 160 Muscidae species and undetermined material of Graphomya. The five putative lineages of Graphomya based on 1.2% threshold are highlighted in different colours. Bootstrap values based on 1000 replicates. [file 1472-6785-12-24-S3.pdf]
